# Exposure to bile influences biofilm formation by *Listeria monocytogenes*

**DOI:** 10.1186/1757-4749-1-11

**Published:** 2009-05-28

**Authors:** Máire Begley, Colm Kerr, Colin Hill

**Affiliations:** 1 Alimentary Pharmabiotic Centre, University College Cork, Cork, Ireland; 2 Department of Microbiology, University College Cork, Cork, Ireland

## Abstract

In the present study we demonstrate that the initial attachment of *Listeria monocytogenes *cells to plastic surfaces was significantly increased by growth in the presence of bile. Improved biofilm formation was confirmed by crystal violet staining, microscopy and bioluminescence detection of a luciferase-tagged strain. Enhanced biofilm formation in response to bile may influence the ability of *L. monocytogenes *to form biofilms *in vivo *during infection and may contribute to survival of this important pathogen in the human gastrointestinal tract and gallbladder.

## Findings

To survive in and subsequently colonize the human gastrointestinal tract the food-borne pathogen *Listeria monocytogenes *must overcome numerous sub-optimal conditions, including exposure to bile in the intestine (reviewed in [1]). Recent research has shown that the bacterium is capable of tolerating high levels of bile *in vitro *and a number of the mechanisms involved have been elucidated [2-5]. *L. monocytogenes *can be isolated from the faeces of asymptomatic healthy humans [6] and *L. monocytogenes *cholecystitis (infection of the gallbladder which is the site of bile storage) in humans has been documented [7,8]. *In vivo *bioluminescence experiments in murine models have revealed that *L. monocytogenes *cells growing in the gallbladder can be secreted *via *bile into the intestine to re-infect the intestinal tract of the same animal or be transmitted in faeces [9]. Bacterial factors involved in colonization of the gallbladder have not yet been identified.

Bile has been shown to affect various properties (such as motility, invasion and toxin production) that may assist the intra-host survival of several enteric bacteria (reviewed in [5]). Bile has also been shown to influence biofilm formation by pathogenic genera (e.g. *Salmonella enterica *var. Typhimurium and *Vibrio cholerae*) [10,11] and indigenous commensal bacteria (e.g. *Bacteroides fragilis *and *Lactobacillus rhamnosus*) [12,13]. Biofilms are surface-associated communities of bacteria embedded in an organized, self-produced extracellular polymeric matrix [14]. The formation of biofilms by *L. monocytogenes *in response to food processing-related environmental conditions has previously been examined and experiments were generally performed at temperatures of 30°C and below [15-19]. The purpose of the present study was to examine the affect of bile exposure on biofilm formation at the physiological temperature of 37°C.

*L. monocytogenes *strain EGDe was grown to early log phase (OD595 nm of ~0.2) in BHI broth (control) and BHI broth containing 0.3% bile (oxgall Sigma B3883) (bile exposed), a concentration which was chosen to approximate the average levels of bile *in vivo *(both media were approx. pH 7.2). Cells were centrifuged (8,000 × g for 6 min) and cell pellets were washed once in 1/4 strength Ringer's solution and re-suspended in fresh BHI broth. Biofilm assays were performed as previously described [16,18] with minor modifications. 100 μl of washed cells were transferred into 10 ml BHI (final concentration of approximately 2 × 10^6 ^cfu/ml) and aliquots were transferred into 96 well microtitre plates (Sarstedt, Cat. No. 82.1581.001), 6 well microtitre plates (Becton Dickinson Cat. No. 353846) or 60 mm Petri dishes (Sarstedt, Cat. No. 82.1194) (200 μl, 3 ml and 4.5 ml, respectively). All plates were sealed with parafilm to prevent evaporation and incubated statically at 37°C. At various time points, the contents of each well were removed, the plates were washed three times with sterile distilled water to remove loosely adhered bacteria, dried at room temperature for 30 min and stained with an aqueous 1% crystal violet solution for 45 min. Excess stain was rinsed off and the dye that was bound to adherent cells was re-solubilised with 96% ethanol. Optical density (OD) was measured at 595 nm using a Beckman DU640 spectrophotometer.

**Figure 1:**
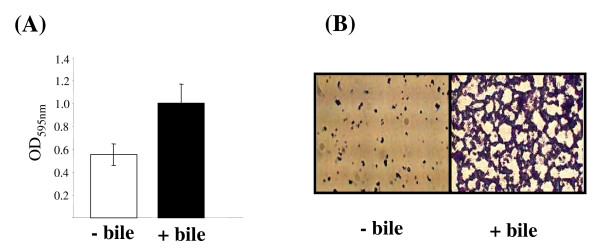
**Biofilm assays**. (A) Biofilm assays in 96 well microtitre plates were carried out as described in the text. Briefly, cells that were grown in BHI alone (- bile) or BHI containing 0.3% oxgall (+ bile) were washed and inoculated at equal cell numbers into fresh BHI broth and 200 μl was added to individual wells of a 96 well plate. After 24 hrs incubation at 37°C biofilms were stained with crystal violet and de-stained using ethanol and the optical density at 595 nm of the alcoholic crystal violet solutions was determined. Data is presented as averages +/- standard deviations for three biological repeats in one experiment. This result is representative of three independent experiments. (B) Representative images obtained from microscopic observations of biofilm on Petri dishes. Bacteria stained with crystal violet were observed under a 40× objective.

In all cases significantly higher OD readings were obtained for cells that had been exposed to bile when compared to control cells. Figure 1A shows the data obtained for a typical 96 well assay. The biofilm formed in 6 well plates and Petri dishes were examined microscopically using a Leica DMLS microscope containing a digital eyepiece (C & A Scientific Co., Inc.). Three random fields were viewed and representative images were captured. At the time point portrayed in Figure 1B control samples showed sparse attachment with separate micro-colonies randomly distributed over the surface. A more developed biofilm was observed for bile-treated samples and micro-colonies had fused to create a mesh-like "web". Biofilm assays were also carried out with five other *L. monocytogenes *strains isolated from a variety of environments (human intestine, food, silage) and all gave similar results to strain EGDe; i.e. cells that were pre-exposed to bile demonstrated increased biofilm formation compared to their control counterparts (data not shown). This indicated that the observed phenomenon was not specific to strain EGDe.

**Figure 2:**
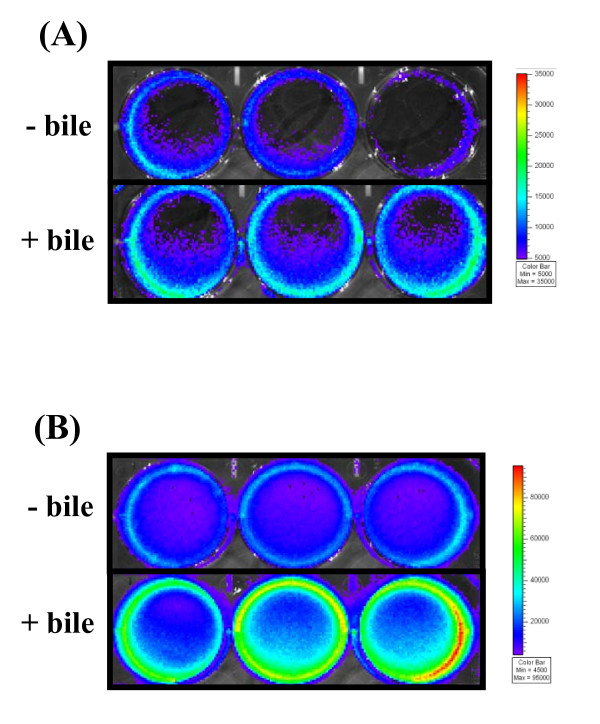
**Bioluminescence imaging of biofilm**. *L. monocytogenes *EGDe *lux *cells that were grown in BHI (- bile) or BHI containing 0.3% oxgall (+ bile) were washed in Ringer's solution and inoculated at equal cell numbers into fresh BHI broth. 3 ml was added to individual wells of a 6-well plate. After (A) 4 hours and (B) 24 hours the contents of wells were removed and loosely attached bacteria were removed by washing with distilled water. 1 ml of fresh BHI broth was added and luminescence was measured in a Xenogen IVIS100. The color bar indicates bioluminescence signal intensity (in photons s^-1 ^cm^-2^). Results shown are those obtained for three biological repeats in one particular experiment and are representative of three independent experiments.

The results of the staining-based biofilm assays were subsequently confirmed in strain EGDe using a bioluminescence-based approach. The entire experiment was repeated exactly as previously described with a constitutively bioluminescent strain *L. monocytogenes *EGDe *lux *(*L. monocytogenes *EGDe transformed with plasmid pPL2 containing P_help _"highly expressed *Listeria *promoter" [20]). Luminescence was measured in relative light units (RLU) (in photons s^-1^) in a Xenogen IVIS100 (Xenogen, Alameda, California, USA). After 24 hours incubation bioluminescence readings for bile-exposed cells were significantly higher than the control (1.64 +/- 0.28 × 10^7 ^RLU versus 1.11 +/- 0.18 × 10^7 ^RLU respectively) (Figure 2B).

Examination of the literature pertaining to the interaction between bacteria and bile allowed us to propose two potential explanations of our observations. Firstly, it has been reported that bile can alter various metabolic pathways of bacteria (reviewed in [5]), raising the possibility that bile-exposed cells may have a higher growth rate than non-exposed cells which could in turn increase the rate of biofilm formation. To examine this hypothesis, the growth rate of bile-exposed and non-exposed cells grown shaking at 37°C in BHI broth was compared by monitoring the optical density at 600 nm (OD_600_) in 96-well plates with a SpectraMax M2 plate reader (Molecular Devices, Sunnyvale, CA). Both exhibited identical growth rates (data not shown).

**Figure 3:**
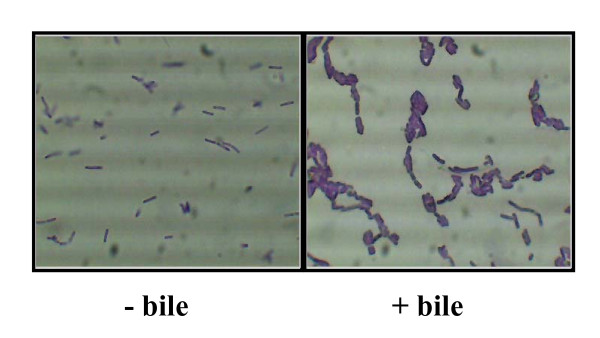
**Microscopy of cells cultured in bile**. *L. monocytogenes *cells were prepared exactly as for biofilm assays (i.e. grown in BHI alone (- bile) and BHI supplemented with 0.3% oxgall (+ bile) and subsequently washed in 1/4 strength Ringers solution). 10 μl of crystal violet was added to 100 μl washed cells in a microcentrifuge tube and mixed. 10 μl was spotted onto slides and viewed with a Leica DMLS microscope containing a digital eyepiece. Three random fields were viewed and representative images were captured.

Secondly, bile has been shown to affect the cell morphology of many bacteria and alterations in membrane characteristics have been reported to affect attachment of cells to inert surfaces [15], (reviewed in [21]). It is well-established that the initial attachment of cells onto a surface is critical for the formation of a biofilm (reviewed in [22]). Higher initial colonization will provide a better base for other cells to attach thereby accelerating the whole process of biofilm development. We therefore examined the morphology of *L. monocytogenes *bile-exposed cells and observed that compared to control cells bile-exposed cells formed longer chains and tended to self agglutinate or co-aggregate (Figure 3). In order to explore the possibility that exposure to bile was affecting attachment to surfaces, biofilm assays were repeated except staining procedures and bioluminescence readings were performed at earlier time points. It was observed that bile-exposed cells attached in higher numbers than control cells. For example, after four hours incubation significantly higher bioluminescence readings were obtained for bile exposed cells (1.05 +/- 0.09 × 10^6 ^RLU) compared to the control (5.88 +/- 1.46 × 10^5 ^RLU)(Figure 2A).

Altogether our experiments demonstrate that exposure to bile results in changes in cell morphology which in turn affects attachment of *L. monocytogenes *resulting in enhanced biofilm formation. Although we have not examined all parameters that may affect biofilm formation *in vivo *(e.g. varying pHs, osmolarities, oxygen tension etc and combinations thereof), and it would be impossible to simulate exact *in vivo *conditions in a laboratory setting, we would like to propose that enhanced biofilm formation in response to bile may improve colonization of the human gastrointestinal tract by *L. monocytogenes *and may also be an important mechanism by which the bacterium can survive in the gallbladder. Biofilm growth may protect bacteria against host defenses and the action of antimicrobial agents but also planktonic cells may be continuously shed from the biofilm to re-infect the same host or be transmitted. It is also possible that *L. monocytogenes *may form biofilm on gallstones in a manner similar to *Salmonella *Typhimurium [11].

In summary, we report our novel observation that exposure to bile affects biofilm formation in *L. monocytogenes*; a finding that may have important implications for the *in vivo *survival of this important pathogen.

## Abbreviations

CFU: colony forming unit; RLU: relative light unit; OD: optical density.

## Competing interests

The authors declare that they have no competing interests.

## Authors' contributions

MB conceived the study, carried out experimental work and drafted the manuscript. CK carried out experimental work and helped to draft the manuscript. CH helped to interpret the data and draft the manuscript. All authors have read and approved the final manuscript.
